# A Comparative Analysis of Genetic and Epigenetic Events of Breast and Ovarian Cancer Related to Tumorigenesis

**DOI:** 10.3390/ijms17050759

**Published:** 2016-05-18

**Authors:** Mckenna Longacre, Nicole A. Snyder, Genevieve Housman, Meghan Leary, Karolina Lapinska, Sarah Heerboth, Amber Willbanks, Sibaji Sarkar

**Affiliations:** 1 Harvard Medical School, Boston, MA 02115, USA; Mckenna_Longacre@hms.harvard.edu; 2 Department of Genetics and Complex Diseases, Harvard T. H. Chan School of Public Health, Boston, MA 02115, USA; nsnyder@hsph.harvard.edu; 3 School of Human Evolution and Social Change, Arizona State University, Tempe, AZ 85281, USA; ghousman@asu.edu; 4 Cancer Center, Department of Medicine, Boston University School of Medicine, Boston, MA 02118, USA; meghanl@bu.edu (M.L.); karolka@bu.edu (K.L.); aw07542@bu.edu (A.W.); 5 School of Medicine, Vanderbilt University, Nashville, TN 37240, USA; sarah.a.heerboth@vanderbilt.edu; 6 Genome Science Institute, Boston University School of Medicine, Boston, MA 02118, USA

**Keywords:** breast cancer, ovarian cancer, epigenetics

## Abstract

Breast cancer persists as the most common cause of cancer death in women worldwide. Ovarian cancer is also a significant source of morbidity and mortality, as the fifth leading cause of cancer death among women. This reflects the continued need for further understanding and innovation in cancer treatment. Though breast and ovarian cancer usually present as distinct clinical entities, the recent explosion of large-scale -omics research has uncovered many overlaps, particularly with respect to genetic and epigenetic alterations. We compared genetic, microenvironmental, stromal, and epigenetic changes common between breast and ovarian cancer cells, as well as the clinical relevance of these changes. Some of the most striking commonalities include genetic alterations of BRCA1 and 2, TP53, RB1, NF1, FAT3, MYC, PTEN, and PIK3CA; down regulation of miRNAs 9, 100, 125a, 125b, and 214; and epigenetic alterations such as H3K27me3, H3K9me2, H3K9me3, H4K20me3, and H3K4me. These parallels suggest shared features of pathogenesis. Furthermore, preliminary evidence suggests a shared epigenetic mechanism of oncogenesis. These similarities, warrant further investigation in order to ultimately inform development of more effective chemotherapeutics, as well as strategies to circumvent drug resistance.

## 1. Classification of Breast and Ovarian Cancer

Globally, breast cancer persists as the most common cause of cancer death in women, and the leading cause of death in women ages 40–49. Despite an over 95% sensitivity of current breast cancer screening, overall mortality has decreased by only 30%. Though less common, ovarian cancer is the fifth leading cause of cancer death in women in the US, owing both to its virulence, and our lack of effective screening. This reflects the continued, if not dire, need for further understanding and innovation in these respective fields of cancer treatment.

This need for more efficacious screening and therapeutics has fueled a rapidly evolving body of scientific work. This has led to an emerging understanding that these clinical entities encompass many heterogeneous cancers, resulting in a new classification schema. Breast cancer tumors were classically categorized by histological origin into infiltrating ductal (76%), invasive lobular (8%), ductal/lobular (7%), mucinous/colloid (2.4%), tubular (1.5%), medullary (1.2%), and papillary (1%). New definitions have simplified the sub groups as luminal A, luminal B, hormone receptor positive and triple negative, which more closely parallels clinical treatment algorithms.

This new schema also aligns with ovarian cancer classification, which includes surface, germ, stromal, and borderline tumors. Of these groups, epithelial carcinomas account for the vast majority (over 90%) and can be divided into serous I, serous II, endometroid, mucinous, Brenner, and clear cell [1]. Based on molecular markers, ovarian tumors can be further classified into Type I *versus* Type II tumors. Type I, or low-grade serous carcinoma, is associated with a ~55% 5 years survival. These tumors develop via the stepwise accumulation of carcinogenic modifications, including BRAF/KRAS. Type II tumors, on the other hand, are often high-grade serous carcinomas (~30% five years survival) at the time of presentation and are associated with various shared patterns of gene expression, as well as frequent p53 mutations. Interestingly, in both breast and ovarian cancer, the vast majority of tumors are of epithelial origin; less commonly, tumors arise from functional and stromal cells. This is further supported by the emerging body of evidence that a significant subset of ovarian cancers in fact originate in the fallopian tubes, adding to the epithelial predominance [1].

## 2. Comparison of the Genetic and microRNA Environments of Breast and Ovarian Cancer

Though breast and ovarian cancer are more often distinct clinical entities, the recent explosion of genomics, proteomics, metabolomics, and other large-scale-omics research, has uncovered many overlaps between the two, including what we and others posit to be relevant shared genetic alterations [2]. These similarities are particularly striking between epithelial-origin triple negative basal cell breast cancer (TNBC) and high-grade serous ovarian cancer (HGS-OvCa). A summary of common mutations in breast and ovarian cancers is listed in Table 1.

**Figure 1:**
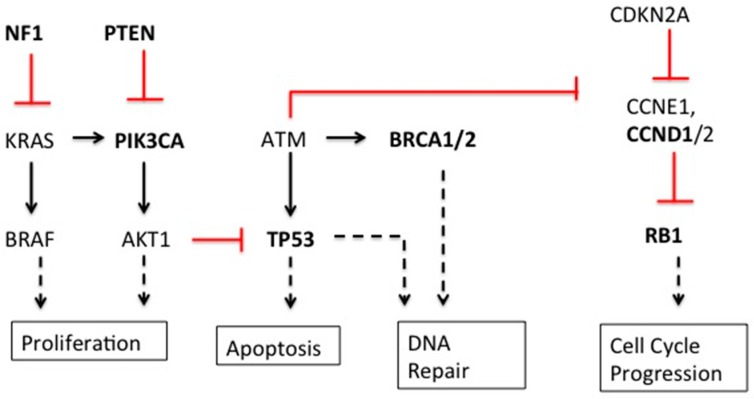
Mutated genes in breast and ovarian cancer. Bolded genes are those significantly mutated in breast and ovarian cancer. Red lines show inhibition of expression; solid black lines show induction of expression; dashed black lines show effector events.

As one might expect, the seemingly disparate genetic alterations are directly or indirectly related to cell cycle control, growth, development, differentiation, DNA damage repair, and apoptosis (Figure 1). Thus, it follows that many of these key genetic alterations have been found in multiple types of cancer, in addition to breast and ovarian [18].

The Human Cancer Genome Atlas, an extensive study investigating gene copy numbers, exon sequences, DNA methylation, and the expression of miRNA, and proteins in breast cancer tumors, identified numerous common genetic alterations among seemingly heterogeneous breast cancers. For example, TP53, PIK3CA, and GATA3 mutations occurred at an incidence greater than 10% across all breast cancers. When separated into molecular subtypes, more commonalities were identified. In basal-like tumors and TBNC (ER-, PR-, HER2-), RB1, BRCA1, and TP53 commonly contained loss-of-function mutations [3]. Interestingly, these mutations are also prevalent in HGS-OvCa tumors [5]. Although mutations in these genes are often found in cancers, the high frequency at which mutations in RB1, BRCA1, and TP53 occur in TNBC and HGS-OvCa may indicate molecular similarities in the development or progression of these two different, aggressive cancers [3].

By far, the most commonly mutated gene in both TNBC and HGS-OvCa is TP53. The Cancer Genome Atlas found that TP53 was mutated in 80% of TNBC tumors and 96% of HGS-OvCa tumors analyzed [3]. Other groups have reported a similarly high prevalence of TP53 mutations in these and other aggressive cancer subtypes [2,3,19] compared to the lower frequency of somatic TP53 mutations seen in many other cancers. For example TP53 mutations have been identified in 27% of brain tumors and 5.8% of cervical cancers [20]. An extensively studied gene, TP53, encodes the transcription factor p53, a protein involved in many signaling pathways that halt cell cycle progression, activate DNA repair mechanisms, and induce apoptosis when activated in response to cellular stress [19,21,22]. Thus, as an anti-proliferative or even pro-apoptotic protein, p53 acts as an important tumor suppressor gene in response to DNA damage. In the context of cancer, it is apparent that tumors with mutations in TP53 are more susceptible to retaining and passing down DNA damage, allowing for the selection of more aggressive tumors. The significance of the shared, high TP53 mutation rate in TNBC and HGS-OvCa is striking and suggestive of shared fundamental features of pathogenesis.

Another commonly mutated gene in breast and ovarian cancers, functional BRCA1 protects the cell from double-stranded DNA damage through homologous recombination. BRCA1/2 mutations are present in 5%–15% of all breast and ovarian cancers [3,4,5] and have been found in other hereditary diseases [23,24,25,26], in the absence of homolous recombination, cells are more vulnerable to genomic instability. Accordingly, cancers containing mutations in BRCA1 have numerous copy number aberrations (CNAs) and are more likely to be TNBC or HGS-OvCa. Interestingly, many BRCA1-mutated TNBC show characteristic patterns in CNAs leading to the classification of BRCA1-mutated cancers as BRCA1-like or non-BRCA1-like TNBC [27,28]. These and other studies comparing copy number landscapes of breast and ovarian cancers have reported several common features between TNBC tumors and HGS-OvCa tumors, including common gains within 1q, 3q, 8q, and 12p (in particular HIF1A and MYC) and loss within 4q, 5q, 8p, and 10q (in particular FOXA1, ER, and PTEN) [28].

## 3. Comparison of the Respective MicroRNA Environments

**Figure 2:**
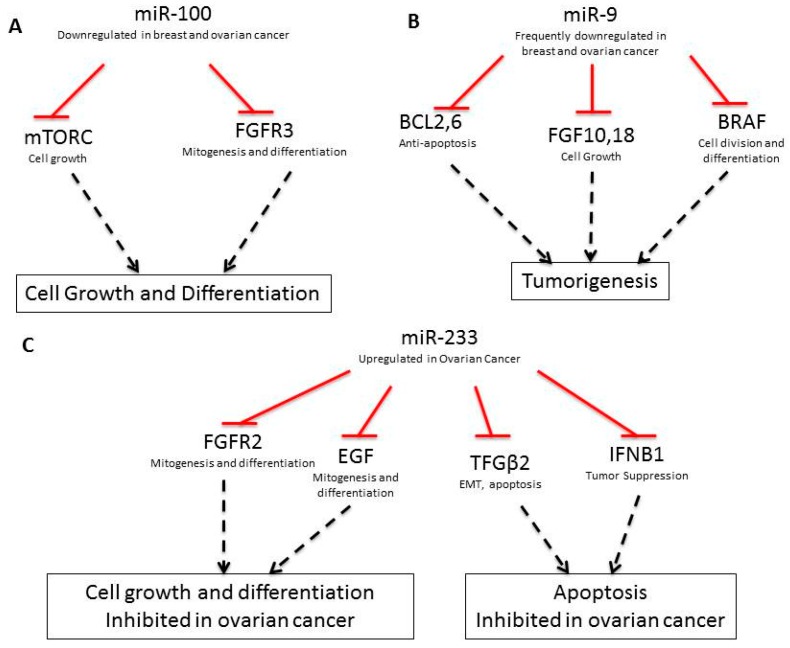
Pathways regulated by miR-100 (**A**), miR-9 (**B**), and miR-233 (**C**). Red lines show inhibition; dashed black lines show effector events.

In addition to coding-DNA mutations, breast and ovarian cancers contain an array of miRNA expression alterations, which contribute to the development and progression of cancer [29]. In healthy cells, miRNA regulate mRNA stability, ensuring proper timing in the formation of functional proteins. Thus, when misregulated, miRNAs can lead to improper protein translation. Table 2 lists misregulated miRNAs implicated in breast and ovarian cancers, respective gene targets and the functional implications in cancer. For example, miR-100, miR-214, miR-206, and miR-233 are involved in diverse pro-growth and anti-apoptotic pathways. miR-100 targets the pro-growth proteins FRAP1/mTOR and FGFR3 for degradation and when downregulated, these genes are re-expressed and function as oncogenes (Figure 2A, [30,31,32,33,34,35,36,37,38,39,40,41,42]). miR-214 deregulation induces cell survival and cisplatin resistance. In breast cancer, downregulation of miR-214 leads to increased EZH2 expression which causes increased cell proliferation [32]. EZH2 is an important epigenetic regulator that positively regulates insulation zone formation for selective gene silencing by enhancing H3K27me3. In ovarian cancer, miR-214 targets PTEN, leading to activation of the Akt cell survival pathway. Akt inhibitors have been found to nullify miR-214 induced cell survival [32]. Interestingly, EZH2 is a part of the polycomb group of proteins that helps to regulate gene expression during normal development. On the other hand, miR-206 is upregulated in breast cancer and targets the ERα gene [41].

As shown in Table 2, several miRNAs are also involved in the increased invasiveness of breast and ovarian cancers and the development of higher grade tumors. For example, the downregulation of miR-9-3, is implicated in breast cancer metastasis, vascular invasion, angiogenesis, and drug resistance development [31,40]. miR-9 expression downregulates BCL2, BCL6, FGFR10, FGFR18, and BRAF, which are involved in anti-apoptosis, cell-growth, and differentiation. Downregulation of miR-9 upregulates these pathway (Figure 2B, [43]).

Additionally, miR-233 downregulates FGFR2 and EGF, which are involved in cell growth and differentiation, as well as TGF-β2 and IFNB1, which are involved in apoptosis. miR-233 is upregulated in ovarian cancer, downregualting these pathways (Figure 2C, [44]). Other miRNAs, including miR-34c, miR-125a, miR-125b, miR-200a, and miR-200c, are downregulated during the epithelial-to-mesenchymal (EMT) transition in ovarian cancer cells [45,46,47,48]. Downregulation of miR-34c, miR-125a, and miR-125b also induces EMT in breast cancer-initiating cells [36]. In particular, miR-34c gene down-regulation via DNA methylation has been shown to promote self-renewal and EMT in breast tumor-initiating cells [36]. Downregulation of miR-125a suppresses ARID3B, a gene which is overexpressed in human ovarian cancer [49]. Furthermore, sequence alternations in miRNAs interact pathologically with BRCA1 and BRCA2. For example, a polymorphism found within the miR-146a sequence has been found to alter the stem region of this miRNA from G:U to C:U. This variant allele produces more mature miR-146a than the wild-type, and the mutant variant has a significantly stronger binding capability to its target BRCA1 and BRCA2 mRNA sequences, which are, themselves, arguably the most studied shared mutations of breast and ovarian cancer [50].

## 4. Epigenetic Comparison of Breast and Ovarian Cancer

In addition to genetic alterations and miRNA, breast and ovarian cancers frequently share key epigenetic patterns. Research has identified consistent methylation patterns that often precede breast cancer progression. For example, moderate to low levels of lysine acetylation (H3K9ac, H3K18ac, and H4K12ac), lysine methylation (H3K4me2 and H4K20me3), and arginine methylation (H4R3me3) were observed in HER2-positive tumors [51]. Similar data with respect to ovarian cancer is still emerging. The level of histone methylation depends on the activation or deactivation of specific histone methylases or demethylases, and alterations of the methylation status of histones has been clearly demonstrated in breast cancer [52,53]. In the case of ovarian cancers, histone splicing is observed. For example, alternative splicing of H2A type histone variants have been observed in ovarian cancers. Additionally, investigations on nuclear dynamics changes are currently underway in ovarian cancer [54] and breast cancer [55]. Importantly, common histone methylation patterns in breast and ovarian cancer include H3K27me3, H3K9me2, H3K9me3, H4K20me3, and H3K4me. In fact, Elisheikh *et al.* [51] have found a consistent correlation between H4K16me3 and 78.9% of all breast cancers. Recent studies showed that mutation of DNA methylases can increase methylation of specific lysine residues in histone 3, causing changes which favor carcinogenesis [56]. For example, the DNA methyltransferase EZH2, which causes H3K27me3, is mutated in breast cancer [56]. Interestingly, H3K27me3 recruits the CCCTC motif binding protein CTCF, which creates an insulation zone and inhibits enhancer and promoter interactions to silence specific genes during development [57,58,59]. These interactions are disrupted by DNA methylation around the interacting sites. For example, in breast cancer, histone modifications at enhancers were observed to regulate genes even up to 750 kilobase. In addition, about 50% of active enhancers were found in nucleosome depleted regions. Expression data analysis identified 600 active enhancers. The genes regulated by these enhancers have functions which include proteolysis, epidermis development, cell adhesion, mitosis, cell cycle, and DNA replications [55].

In addition to histone modifcations, global modification of DNA sequences by methylation at CpG residues plays an important role in oncogenesis of both breast and ovarian cancer. As described above, methylation of the enhancer sites reverse CTCF regulated DNA silencing [60], but methylation of specific CpG islands in the promoter regions of genes silence them [61,62]. Shared targets of methylation include the promotor regions of p21, p16, MGMT, BRAC1, MLH1, HOXD11, CDH1 (E-cadherin), TGF-R, ARHI, and RASSF1A in breast and ovarian cancer [63].

In the case of breast cancer, 30% of tumors are associated with an overexpression of HER2 due to amplification of gene copy number [64]. Interestingly, overexpression of HER2-mediated signaling has been shown to alter the genome-wide methylation pattern [65,66]. Specifically, the genes most commonly methylated in these tumors were relevant to development and transcription (AKT3, HK1, PFKP, AKR1B1, INA, FOXC2, NEUROD1, CDKL2, IRF4) or were Homeobox genes (DBX1, NX-6, SIX6) [67]. Other well-known drivers of breast cancer metastasis include upstream methylation of E-cadherin, RARRF1A, RAR-2, APC, TWIST, and GSTP1 [68]. Another important example, cell adhesion receptor integrins including α5β6, may also regulate methylation in breast cancer cells [69,70]. Interestingly, it has been shown that HER2 and integrin receptors associate to enhance downstream signaling for breast cancer progression [71]. It is reasonable to believe that these downstream signaling processes regulate DNA methylation. In support of this notion, a recent study showed that ERK kinase regulated DNA Methyltransferase 1 (DNMT1) and methylation of specific genes in prostate cancer cells [72]. In addition to ERK, Akt DNA methylation also regulates H3K27me3 mediated gene silencing [73]. This suggests that intracellular signaling plays a significant role in the methylation process regulating gene silencing. Usually, DNMTI is highly expressed in cancer cells compared to normal cells where its expression varies at different stages of the cell cycle [74]. A recent system biology study showed that higher expression of DNMTI does not necessarily methylate and silence all types of genes in cancer cells. DNMTI is allosterically activated at the locations of genes it silences [75]. Taken together, this suggests a complicated but well-orchestrated mechanism by which methylation and thereby gene silencing are regulated. Changes in the balance can, thus, perturb epigenetic regulation and gene expression.

**Figure 3:**
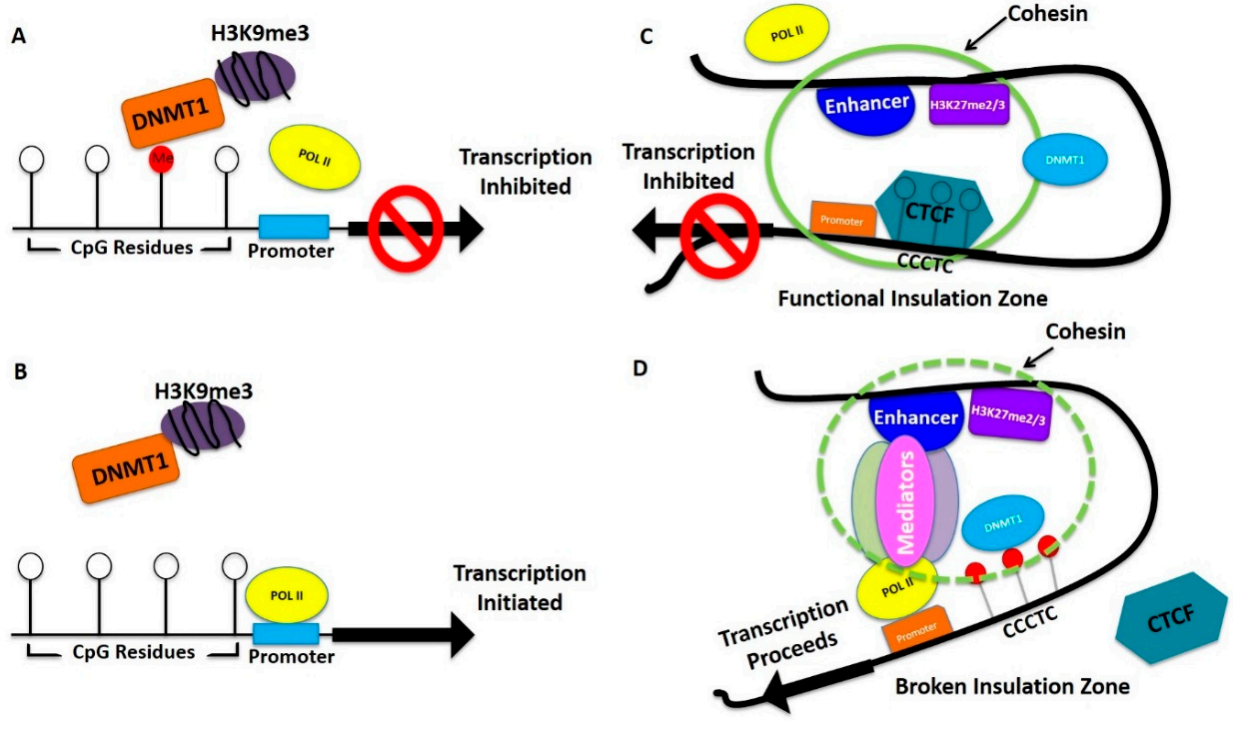
Model of DNA methylation and histone methylation regulating gene expression for breast and ovarian cancer initiation. (**A**,**B**) Hypermethylation is indicated by red balls (CpG residue). White balls represent unmethylated CpG residues. DNMT1 represents DNA methyl transferase 1, POLII represents RNA polymares II; (**B**) In the absence of hypermethylation, POLII is able to bind to the promoter region and initiate transcription; (**C**,**D**) White balls represent unmethylated, and red balls represent hypermethylated CpG residues. CCCTC is CTCF binding motif. The green circle represents effective insulation zone. Break of insulated zone is shown by dotted green circle.

Figure 3 depicts a simplistic model of possible epigenetic regulation of tumor suppressor genes and growth promoting genes in breast and ovarian cancer progression. Many of the epigenetically-regulated genes are common in both types of cancers and some are different but the regulation could be similar. Hypermethylation of promoter regions blocks POLII binding inhibiting transcription of tumor suppressor genes (Figure 3A). As this process is reversible, when these regions are demethylated by treatment with epigenetic drugs, POLII binds and tumor suppressor genes are re-expressed (Figure 3B), which is an important aspect of current improvement as combination therapy. In contrast, growth promoting genes are kept under controlled expression by CTCF binding to the H3K27me2/me3 regions creating an insulation zone and inhibiting enhancer-promoter interaction necessary for POLII binding and transcription initiation (Figure 3C). In both breast and ovarian cancer this control could be lost because of hypermethylation around the region where CTCF binds (Figure 3D), which causes an above normal expression of growth promoting genes. Demethylation as a result of epigenetic drug treatment, allows CTCF to bind in these regions controlling above normal growth promoting gene expression. Since the formation of breast cancer or ovarian cancer progenitor cells is a long term process, gradual epigenetic changes which cause alteration of gene expression slowly convert a few pre-disposed cells to breast or ovarian cancer progenitor cells. Other genetic alterations help advance and speed up this progression. Overall, this model shows that a change of balance in CpG DNA methylation in upstream promoter regions and in enhancers along with differential histone methylations regulates the initial steps in tumorigenesis. Although not currently included in this model, histone modifications, such as H3K4, are known to facilitated gene expression. Additionally, lnc RNA may also have a role in this process [76]. However, future studies will reveal how much these factors contribute to the described model.

## 5. Role of Stroma in Breast and Ovarian Cancer

Epigenetic regulation originates both intracellularly and extracellularly. Stromal cells are central to this process, providing extracellular signals, and inducing intracellular changes via cell-cell interactions. As described above, signaling pathways (e.g., ERK and AKT) regulate methylation. Cytokines, growth factors, and hormones from the extracellular milieu, as well as direct attachment of stromal cells to breast or ovarian epithelial cells, regulate intracellular signaling. The relationship between stromal mutations and carcinogenesis is well known [14,68,77]. Furthermore, ovarian cancers have been associated with higher numbers of fibroblasts, lymphocytes and other inflammatory cells. These cell types may receive deranged regulatory signals from surrounding pathological cells. For example, it has been shown that ovarian surface epithelial cells that have been immortalized by Ras may reprogram the stromal microenvironment through the senescence of fibroblasts, and through this mechanism, support tumorigenesis [77]. Furthermore, expression of the chemokine CXCR4 has been found in 59% of ovarian cancers, and CXCL12 in 91% of ovarian cancers; both have been associated with decreased disease free survival [78]. Finally, as with tumor genomes, studies have shown that methylation levels are elevated in oncogenic breast stromal environments, often even before there are detectable tumors [79]. Taken together, these examples support the notion that stromal changes produce alterations in signaling that lead to increased methylation and histone modifications, which in turn drives the formation of cancer progenitor cells (Figure 3). Although breast and ovarian stromal environments are morphologically and functionally distinct, there are many shared common epigenetic signaling pathways. Furthermore, as previously discussed, continued study of the fallopian (epithelial) origin of various ovarian cancers my further our understanding of the pathogenicity of the respective stromal environments.

## 6. Current and Future Treatment of Breast and Ovarian Cancer

In the case of breast cancer, clinical management is primarily driven by the hormonal and receptor status of the tumor, as well as stage and grade. At this time, the standard of care necessitates genetic testing of all breast cancer tumors. In both hormone receptor positive and negative tumors, standard treatment begins with surgical excision, then local radiation, and finally chemotherapy guided by the specific genetic diagnosis. Common agents include Tamoxifen (estrogen receptor antagonist), Herceptin (HER2 inhibitor), and proteasome inhibitors. Thus, in breast cancer, depending on the origin or the classification, target-specific drugs are part of the standard of care. In patients that have an underlying predisposition to cancer, prophylactic surgery may also be considered.

The clinical manifestations of ovarian cancer are distinct among the gynecological malignancies in that there is no standard screening protocol, and at best, subtle clinical signs; diagnosis can only be confirmed by surgery. As such, at the time of presentation, the vast majority of neoplasms have metastasized beyond the pelvis (grade III). Management includes surgical debulking, followed by systemic chemotherapy. Since most cancers have metastasized before presentation, local radiation is not the norm, except in select clinical scenarios including noninvasive tumor subtypes such as benign teratomas. Standard regimens often include an intercalating agent, an alkalating agent (e.g., cis-platin, carboplatin), an anti-growth agent (e.g., vincristine) and in some cases an anti-tetrahydrofolate reductase (e.g., methotrexate). In contrast to breast cancer, chemotherapies used in the management of ovarian cancer are often not target specific.

## 7. Drug Resistance

One of the major problems associated with breast and ovarian cancer chemotherapeutics is the development of drug resistance. Clinically, this manifests as frequent relapse, and even more importantly, relapse of tumors of higher virulence and intractability. For example, although most women with advanced stage ovarian cancer initially respond to cytoreductive surgery and platinum based chemotherapy, over 70% of women relapse. Moreover, platinum-resistant epithelial ovarian cancer is considered uniformly fatal [79]. There are numerous pathways by which tumors may gain drug resistance, including efflux by *p*-glycoproteins (MDR gene overexpression), rapid drug metabolism, and alteration of the target site. In addition to traditional selective mechanisms, recent studies have indicated that methylation plays a significant role in this process [80,81,82]. For example, ER-i has been associated with tamoxifen resistance, and is methylated in 50% of breast cancers [83,84]. Methylation of the MCJ gene has been associated with poor response to therapy, and poor overall survival [85]. Demethylation of Fanconi anemia complementation group F (FANCF) with decitabine has been shown to decrease sensitivity to cisplatin in various cell models [15]. Methylation of hMLHI (a DNA mismatch repair protein) and subsequent loss of expression is associated with resistance to cis-platin [85]. Importantly, demethylation and, thus, reversal of this and other pathological modifications, has been associated with resensitization to therapy [86]. These various examples are supported by the more general observation that DNMT1 inhibitors (AZA derivatives) and histone deacetylase inhibitors (HDACi) have been shown to resensitize resistant ovarian cancer cells to cisplatin by demethylation and re-epression of the RGS10 [87,88]. Though epigenetic changes are observed in breast and ovarian cancers, no systematic study has been performed to compare the global methylation status before *versus* after remission of both types of cancers.

Drawing inferences from similar studies in leukemia patients in which methylation levels remained high throughout treatment, it is likely that a durable chemotherapeutic response will require treatment that includes agents that modify global methylation and, thus, attack cancer progenitor cells [61,81,84,89,90]. Furthermore, these agents must have the capacity to modulate chromatin opening, as well as inhibit CpG methylation, in order to render drug resistant cells vulnerable to treatment, while at the same time inhibiting the formation of new cancer progenitor cells. In support of this notion, it has been shown that HDAC inhibitors (Class I and Class III) possesses the ability to successfully perform both of these functions [90]. When these agents are combined with traditional chemotherapeutics, even in suboptimal doses, they produce additive, or even synergetic death of breast and ovarian cell lines [91,92].

Several clinical trials of such combination therapies are underway. It has been demonstrated by these authors and others that the efficiency of demethylation increases when HDACi are used in combination with DNMT1i [61,88,89,90,91,92,93]. Others have shown that such combinations are highly effective against breast cancer in mouse models [94]. Furthermore reversal of platinum drug resistant ovarian cancer after treatment with HDACi [88], and similarly reduction in the relapse of lung cancer patients with use of epigenetic chemotherapeutics, have been reported [95]. As chemotherapy of ovarian cancer is mainly target non-specific, and because epigenetic modifications possibly play similar types of roles in ovarian and breast cancers, current investigators are extremely encouraged to develop epigenetic therapies in combination with traditional drugs for breast and ovarian cancers. [50,61,62,91,92,94,96,97,98,99].

## 8. Inferences from a Comparison of Breast and Ovarian Cancer: An Epigenetic Link

Research continues to reveal that breast and ovarian cancer have a surprising number of shared genetic, stromal, and epigenetic features, despite profoundly different clinical presentations. This has led us to consider shared features of pathogenesis. Amidst the seemingly profound heterogeneity of breast cancers, it has been suggested that all solid tumors may originate from 2 to 3 types of breast cancer stem cells [2,3,100]. This is at odds with the classical notion that breast cancer development is driven from myriad distinct genetic alterations. Rather, it is suggestive of a shared pathogenic mechanism, which is then followed by the generation of diverse passenger mutations. There is a significant body of indirect evidence that the shared mechanism for these discrete cancer events is epigenetically driven. For example, methylation increases in breast and ovarian stromal cells prior to carcinogenesis. This may suggest that oncogenesis is driven by expression of stromal signaling molecules, which in turn induces epigenetic changes in target breast or ovarian cells, as described in previous sections [50,101]. Furthermore, it has been reported that cancer progenitor cells are only irradiated when treated with epigenetic modulating drugs [2]. Therefore, further research is likely to reveal that the most significant commonalities between breast and ovarian carcinogenesis are shared anteceding and perhaps causational patterns of epigenetics.

## Figures and Tables

**Table 1:** Comparison of significant genetic alterations in breast and ovarian cancers.

Gene	Type	Gene Function	Breast	Ovarian
BRCA1/2 [3,4,5]	Mutation	DNA homologous recombination repair	Yes	Yes
TP53 [1,3,5]	Mutation	Cell cycle checkpoint	Yes	Yes
RB1 [3,5]	Mutation/Deletion	Cell cycle regulator	Yes	Yes
NF1 [3,5]	Mutation/Deletion	Negative regulator cell division via Ras inhibition	Yes	Yes
FAT3 [3,5,6]	Mutation	Central nervous system development	Yes	Yes
CSMD3 [3,5,7]	Mutation, Copy Number	Development	-	Yes
GABRA6 [5]	Mutation	GABA receptor, neurons	-	Yes
CDK12 [5,8]	Mutation	RNA splicing regulation	-	Yes
BRAF [5]	Mutation	Proto-oncogene, cell growth signals	-	Yes
PIK3CA [3,5]	Mutation	Cell growth, Catalytic subunit of PI3k, signaling cascades including activation of Akt	Yes	Yes
KRAS [5]	Mutation	Cell growth, Signal propagation including growth factor and PI3k signals	-	Yes
NRAS [5]	Mutation	Cell growth, Signal propagation including growth factor and PI3k signals	-	Yes
CCNE1 [5,9]	Copy Number Amplification	Cycle E1—cell cycle regulation	-	Yes
MYC [3,5]	Copy Number Amplification	Txn factor, involved in cell cycle progression and apoptosis	Yes	Yes
MECOM [5,10]	Copy Number Amp	Differentiation, apoptosis, stem cell quiescence [6]	-	Yes
ZMYND8 [5,11]	Copy Number Amp	C-kinase receptor, possibly involved in DNA damage recognition [7]	-	Yes
IRF2BP2 [5]	Copy Number Amp	P53 target	-	Yes
ID4 [5,12]	Copy Number Amp	Transcription inhibition development, growth differentiation, senescence, apoptosis, angiogenesis	-	Yes
PAX8 [5,13]	Copy Number Amp	Development	-	Yes
TERT [5]	Copy Number Amp	Telomerase, genome stability	-	Yes
PTEN [3,5]	Deletion	cell cycle and apoptosis, possibly migration, adhesion, and angiogenesis	Yes	Yes
CREBBP [5]	Deletion	Cell cycle control		Yes
AKT1 [3]		Apoptosis	Yes	-
GATA3 [3,14]	Mutation	Differentiation of luminal cells, Estrogen Receptor pathway	Yes	-
CDH1 [3]	-	Cell adhesion, cell cycle regulation	Yes	-
MLL3/KMT2C [3,15]	-	Demethylation of H3K27, differentiation	Yes	-
MAP3K1 [3]	-	MAPK/ERK pathway-cell cycle	Yes	-
CDK1B [3]	-	Cell cycle progression	Yes	-
TBX3 [3,16]	Mutation	Mammary gland development	Yes	-
RUNX1 [3]	-	Development and differentiation, hematopoiesis	Yes	-
CBFB [3]	-	Development, stem-cell homeostasis	Yes	-
AFF2 [3,5,17]	-	Cell proliferation	Yes	Yes
PTPN22 [3]	-	Immune signaling, responsiveness of T and B cells	Yes	-
PTPRD [3]	-	Cell cycle, growth, differentiation	Yes	-
SF3B1 [3]	-	Splicing	Yes	-
CCND3 [3]	-	Cell cycle	Yes	-

**Table 2:** miRNA implicated in breast and ovarian cancers.

miRNA	Up-/Downregulated	Gene Target	Gene Activity	Breast Cancer?	Ovarian Cancer?	Function
miR-100 [29]	Down	FRAP1/mTOR, FGFR3 [30]	-	Yes	Yes	Cell growth and survival
miR-9 [31]	Down	FGF18, FGF10, BCL2, BCL6, BRAF, CLDN14, CLDN6, SEPTIN10, ZNF, PVRL2, LASS4, BCL2, CLDN, FGF	-	Yes (miR-9-3)	Yes	Drug resistance
miR-214 [29,32]	Down	PTEN, EZH2	Suppression	Yes	Yes	Cell survival, cisplatin resistance, Akt
miR-125a [33]	Down	HER2, ARI3B	Suppression	Yes	Yes	EMT
miR-125b [33]	Down	HER2	Suppression	Yes	Yes	EMT
miR-22 [34,35]	-	miR-20	-	Yes	-	Metastasis
miR-34c [36]	Down	-	-	-	-	EMT
miR-199a [37]	Down	-	-	-	Yes	
miR-200a [38]	Down(EMT)/Up(MET)	ZEB2	Suppression	-	Yes	EMT
miR-200c [38]	Down(EMT)/Up(MET)	ZEB1/2	Suppression	-	Yes	EMT
miR-146a	Up (variant allele)	BRCA1/2	-	-	Yes	-
miR-210 [39]	Down (CNA)	E2F3 (TxF)	-	-	Yes	HIF
miR-233 [40]	Up	FGFR2, EGF, S100A3, KRAS, TGFΒ2, IFNBI, SPINKS, E2F1, SEPTIN6, MMP9, USF2	-	-	-	Ras, integrin signal
miR-206 [33,41]	Up	ERα	Suppression	Yes	-	-

## Abbreviations

AKR1B1	Aldo-keto reductase family 1, member B1
APC	Adenomatous polyposis coli
ARHI	Aplysia ras homology member I (GTP binding protein Di-RAS3)
ARID3B	AT-rich interactive domain-containing protein 3B
CBFB	Core-binding factor subunit beta
CDH1	Cadherin-1
CDK	Cyclin kependent kinase
CDKL2	Cyclin-dependent kinase-like 2
CREBBP	CREB-binding protein
DBX1	developing brain homeobox protein 1
DNMT	DNA methyltransferase
DNMTi	DNA methyltransferase inhibitor
EGF	Epidermal growth factor
EMT	Epithelial mesenchymal transition
ER	Estrogen receptor
ER-	Estrogen receptor negative
EZH2	Enhancer of zeste homolog 2
FANCF	Fanconi anemia complementation group F
FGF	Fibroblast growth factor
FGFR	Fibroblast growth factor receptor
FRAP1	FK506-binding protein 12-rapamycin-associated protein 1 (Mammalian target of rapamycin, MTOR)
GABRA6	Gamma-aminobutyric acid receptor subunit alpha-6
GSTP1	Glutathione S-transferase P
HDACi	Histone deacetylase inhibitor
HGS-OvCa	high-grade serous ovarian cancer
HIF	Hypoxia induced factor
HK1	Hexokinase-1
IRF2BP2	Interferon regulatory factor 2-binding protein 2
IRF4	interferon regulatory factor 4
KMT2C	Lysine *N*-methyltransferase 2C (Mixed-lineage leukemia protein 3, MLL3)
MAP3K1	(Mitogen-Activated Protein Kinase Kinase Kinase 1
MDR	Multidrug resistant
MECOM	MDS1 and EVI1 complex locus protein EVI1
MGMT	*O*-6-Methylguanine-DNA methyltransferase
miR	microRNA
miRNA	microRNA
MLH1	MutL homolog 1
MMP9	Matrix metallopeptidase 9
NEUROD1	Neuronal differentiation 1
NF1	Neurofibromin 1
PAX8	Paired box gene 8
PFKP	Phosphofructokinase, platelet
PIK3	Phosphatidylinositol-4,5-bisphosphate 3-kinase
PTEN	Phosphatase and tensin homolog
PTPRD	Protein tyrosine phosphatase, receptor type D
PVRL2	Poliovirus receptor-related 2
RASSF1A	Ras association domain-containing protein 1
RB1	Retinoblastoma 1
RUNX1	Runt-related transcription factor 1
SF3B1	Splicing factor 3B subunit 1
TERT	Telomerase reverse transcriptase
TGF	Transforming growth factor
TNBC	triple negative basal cell breast cancer
TxF	Transcription factor
USF2	Upstream stimulatory factor 2
ZEB	Zinc finger E-box-binding homeobox 1
ZMYND8	Protein kinase C-binding protein 1
